# Influence of body weight, age, and sex on cerebrospinal fluid peak flow velocity in dogs without neurological disorders

**DOI:** 10.1111/jvim.17073

**Published:** 2024-04-25

**Authors:** Johannes Rich, Sarah Hubler, Beatriz Vidondo, Mathieu Raillard, Daniela Schweizer

**Affiliations:** ^1^ Division of Clinical Radiology, Departement of Clinical Veterinary Medicine, Vetsuisse Faculty University of Bern Bern Switzerland; ^2^ Veterinary Public Health Institute University of Bern Liebefeld Switzerland; ^3^ Division of Anesthesiology and Pain Management, Departement of Clinical Veterinary Medicine, Vetsuisse Faculty University of Bern Bern Switzerland

**Keywords:** canine, cerebrospinal fluid, MRI, phase‐contrast

## Abstract

**Background:**

Changes in the brain can affect the flow velocity of cerebrospinal fluid (CSF). In humans, the flow velocity of CSF is not only altered by disease but also by age and sex. Such influences are not known in dogs.

**Hypothesis:**

Peak flow velocity of CSF in dogs is associated with body weight, age, and sex.

**Animals:**

Peak flow velocity of CSF was measured in 32 client‐owned dogs of different breeds, age, and sex.

**Methods:**

Peak flow velocity of CSF was determined by phase‐contrast magnetic resonance imaging (PC‐MRI) at the mesencephalic aqueduct, foramen magnum (FM), and second cervical vertebral body (C2). Dogs were grouped according to body weight, age, and sex. Flow velocity of CSF was compared between groups using linear regression models.

**Results:**

Dogs with body weight >20 kg had higher CSF peak velocity compared with dogs <10 kg within the ventral and dorsal subarachnoid space (SAS) at the FM (*P* = .02 and *P* = .01, respectively), as well as in the ventral and dorsal SAS at C2 (*P* = .005 and *P* = .005, respectively). Dogs ≤2 years of age had significantly higher CSF peak flow velocity at the ventral SAS of the FM (*P* = .05). Females had significantly lower CSF peak flow velocity within the ventral SAS of FM (*P* = .04).

**Conclusion:**

Body weight, age, and sex influence CSF peak flow velocity in dogs. These factors need to be considered in dogs when CSF flow is quantitatively assessed.

**Abbreviations**
C2: second cervical vertebral body
CSF: cerebrospinal fluid
FM: foramen magnum
FOV: field of view
NSA: number of signal averages
PC‐MRI: phase‐contrast magnetic resonance imaging
ROI: region of interest
SAS: subarachnoid space
TE: echo time
TR: repetition time
VENC: velocity encoding

## 1 INTRODUCTION

On one hand cerebrospinal fluid (CSF) flow is a result of continuous production of CSF, which leads to bulk flow and on the other hand a result of transmitted choroidal arterial pulsations. The latter result in a pulsatile back‐and‐forth motion of CSF that is caused by variation in the intracranial blood volume during the cardiac cycle.^1^, ^2^ During systole, CSF flow caudally to the spinal subarachnoid space (SAS), whereas it moves toward the cranium during diastole.^3^ Changes in CSF flow dynamics have been identified in diseases such as hydrocephalus and Chiari malformation in humans^4^ and Chiari‐like malformation in dogs.^5^

Pulsatile CSF flow can be visualized and quantified by phase contrast magnetic resonance imaging (PC‐MRI).^3^, ^6^, ^7^, ^8^ After the initial radiofrequency pulse, PC‐MRI uses a bipolar gradient applied in the direction of flow.^4^ This causes moving spins to be subjected to a phase shift that is proportional to flow velocity.^4^, ^6^, ^7^, ^8^ The bipolar gradient determines the maximum velocity corresponding to a phase shift of 180°.^6^, ^7^ This maximum velocity is referred to as the encoding velocity (VENC). The VENC is inversely related to the strength of the gradient.^4^ Any measurement that exceeds a phase shift of 180° will result in aliasing.^4^ However, if the VENC is set too high, spatial resolution deteriorates.^4^ Therefore, VENC setting is crucial for proper performance of PC‐MRI and is selected depending on the suspected flow velocity.^4^

The application of PC‐MRI in veterinary medicine has been shown in clinically healthy Beagle dogs at the mesencephalic aqueduct, foramen magnum (FM), and cervical spine.^9^, ^10^ Compared with normal beagle dogs, CSF flow velocity measured in Cavalier King Charles Spaniels diagnosed with syringomyelia was significantly lower at the dorsal aspect of the SAS at the level of intervertebral junction between the second and third cervical vertebral bodies.^5^ Flow velocity of CSF in healthy human subjects decreases with age, and males have higher CSF flow velocities compared with females.^11^, ^12^, ^13^ The influence of age, body weight, and sex on CSF flow velocity has not been investigated in dogs.

Our aim was to compare CSF flow velocity in dogs without structural brain abnormalities and of different breed, age, and sex. We hypothesized that CSF flow velocity would be associated with body weight, age, and sex.

## 2 MATERIALS AND METHODS

Our prospective cross‐sectional study was approved by the Cantonal Veterinary Office of Bern TVB Nr.: BE 4/18.

### 2.1 Animals

Client‐owned dogs presented to the small animal clinic of the Vetsuisse Faculty of Bern were recruited for the study. Neurologically normal dogs that required general anesthesia (eg, orthopedic imaging, ear disease, or implant removal) were included. Exclusion criteria were an abnormal neurological examination, a neurological abnormality mentioned in the medical history of the patients, or a structural brain abnormality detected on magnetic resonance imaging (MRI). All owners signed an informed consent to participate in the study.

For the study, 46 dogs were recruited in 22 months. Eight dogs were excluded because of abnormal neurological examination and 6 dogs because of neurological abnormality mentioned in the medical history. Thirty‐two dogs with a mean age of 3.86 years (median, 2.08 years; range, 0.35‐11.48 years) and a mean body weight of 24.25 kg (median, 23 kg; range, 4‐53 kg) were included. Sixteen of the dogs were females and 16 were males. Three of the dogs were Labrador Retrievers. There were 2 each of Jack Russell Terrier, American Staffordshire Terrier, Great Swiss Mountain Dog, Dachshund, Hovawart, and Border Collie. The remaining dogs were each represented by 1 breed, namely Great Dane, Beauceron, Australian Shepherd, German Shepherd mixed breed, Alaskan Husky, American Bulldog, Briard, English Setter, German Longhaired Pointer, Swiss Scent Hound, Nova Scotia Ducking Toller Retriever, German Spaniel, Springer Spaniel, Cocker Spaniel, Keeshond and Yorkshire Terrier. Four of the 32 dogs were considered brachycephalic breeds.

### 2.2 Anesthesia

Animals were fasted for a minimum of 6 hours. A complete physical examination was performed before the procedure by the attending anesthetist. Dogs received IM methadone (0.2 mg/kg; Methadon Streuli, Streuli Pharma AG, Switzerland) mixed with dexmedetomidine (1‐10 μg/kg, depending on the behavior of the animal; Dexdomitor, Provet AG, Switzerland). Ten minutes later, an IV catheter was placed into a cephalic vein (jelco, 18‐22G, Smiths Medical ASD, Inc, USA).

Dogs were preoxygenated for 3 minutes using a face mask and an oxygen flow of 2 L/minute. Anesthesia then was induced with propofol titrated to effect (Propofol 1% MCT Fresnius, Fresnius Kabi, Switzerland). Dogs were intubated with an orotracheal tube and connected to the anesthetic machine using a rebreathing system (Aespire View, Datex‐Ohmeda, USA). Anesthesia was maintained with sevoflurane (Sustane Sevoflurane, Piramal Critical Care, USA) administered to effect in a mixture of oxygen and air (with an inspired fraction of oxygen of 60%). During the initial procedure, dogs were allowed to breathe spontaneously. However, during the MRI, they were mechanically ventilated. Volume‐controlled ventilation was used with a tidal volume of 10 mL/kg. The respiratory rate was adjusted to maintain an end‐tidal CO_2_ between 35 and 40 mm Hg. Dogs were monitored clinically throughout the procedure (MRI and previous intervention). In addition, instrumental monitoring included pulse oximetry (saturation and pulse rate), heart rate, noninvasive blood pressure, capnography, analysis of the inhalant gas, and spirometry. Plasma‐Lyte solution (Plasma‐Lyte A, Baxter AG, Switzerland) was administered during anesthesia at a rate of 5 mL/kg/hour. Any anesthetic complication (eg, hypotension, bradycardia) was managed on a case‐by‐case basis. An anesthetist continuously monitored the recovery period.

### 2.3 Magnetic resonance imaging

All dogs were examined in a 1.0 T MR unit (Philips, High Field Open Panorama, Philips Medical Systems, the Netherlands). The dogs were positioned in sternal recumbency, with the head in a knee coil (knee coil, Philips Medical Systems, the Netherlands) or head coil (SENSE head coil, Philips Medical Systems, the Netherlands), depending on the size of the head. The head was placed in an extended position with an angle of head position of equal or ≥0°.^14^ Compression of the jugular veins was prevented by supporting the head, if necessary. For pulse‐gated acquisition, an MRI‐compatible peripheral pulse unit (Invivo Corporation, Philips Medical Systems, USA) was placed on the tongue or paw.

**FIGURE 1:**
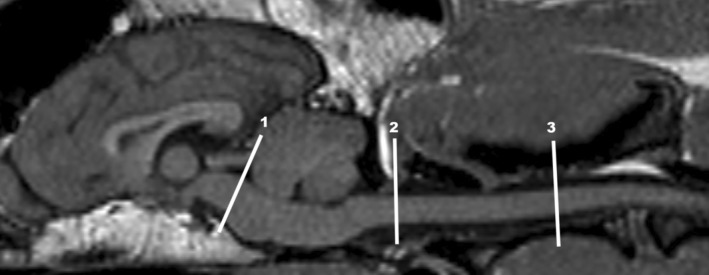
T1‐weighted midsagittal MR image of the brain and upper cervical spine of a dog. The 3 locations of CSF flow measurements are marked with a white line: (1) widest lumen of the mesencephalic aqueduct, (2) foramen magnum (FM) and (3) middle of the spinous process of the second cervical vertebral body (C2). All measurements are aligned perpendicular to the suspected CSF flow.

A sagittal 3D‐T1 weighted sequence (repetition time [TR], 25 ms; echo time [TE], 6.91 ms; flip angle, 30°; field of view [FOV], 160 × 92 × 60 mm; voxel size, 1 × 1 × 1 mm; number of signal averages [NSA], 2) was performed. This allowed the planning of the 3 phase‐contrast sequences and excluded structural brain abnormalities. Transverse phase contrast images were planned perpendicular to the assumed CSF flow direction at 3 locations: (a) the widest lumen of the mesencephalic aqueduct, (b) 1 mm caudal to the occipital condyles (FM), and (c) at the middle of the spinous process of the second cervical vertebral body (C2; Figure 1). The parameters for the phase‐contrast sequences were: TR, 17 to 18 ms; TE, 11 to 12 ms; flip angle, 25°; FOV, 80 × 80 mm; slice thickness, 5 mm; voxel size, 0.6 × 0.81 × 5.00 mm; NSA, 2; heart phases, 32; cardiac gating, retrospective. For measurement at the FM and C2, the VENC was a standard setting 5 and 3 cm/s at the level of the mesencephalic aqueduct.

Acquired phase contrast images were qualitatively assessed for the presence of a bright to dark shift in the pixels, where CSF was expected. If no change in the brightness of the pixels appeared, image planning was reevaluated. Furthermore, quality control on the choice of VENC was performed. The sequence was repeated with adjusted lower VENC planning when the color shift remained absent. If aliasing occurred within the CSF space, the scan was repeated, with higher VENC until no aliasing occurred.

The PC‐MRI sequence acquisition time varied with heart rate, and acquisition of the 3 measurements ranged between 15 and 20 minutes.

### 2.4 Analysis of the phase contrast images

The CSF peak flow velocity was calculated based on phase contrast images as well as magnitude images using dedicated software (Extended MR WorkSpace 2.6.3.5, Philips Medical Systems, 2013, the Netherlands). For each location, regions of interest (ROI) were manually drawn within the CSF spaces by the first author who was instructed by a board‐certified radiologist. At the mesencephalic aqueduct, 1 ROI was drawn. At the FM and C2, 3 ROIs were placed: in the dorsal SAS and the right and left ventral SAS to spare the spinal artery.^9^ The mean value of the right and left ventral SAS are expressed as ventral SAS. A time velocity curve and CSF peak flow velocity in cm/s were automatically calculated by the software. The CSF peak flow velocity was chosen for the statistical analysis to avoid partial volume effect.^15^, ^16^ The manual drawing of all ROIs was repeated 3 times by the first author at least 1 week apart. The mean of the CSF peak flow velocity was used for statistical analysis. Phase contrast images where no bright to dark shift in the CSF space was visible or where the time velocity curve did not show the typical bidirectional flow were excluded from the analysis.^9^

### 2.5 Statistical analysis

Body weight and age were analyzed both as numerical and categorical variables. Dogs were classified by body weight (<10 kg, 10‐20 kg, >20 kg). The age was categorized initially into 6 categories: puppy (0‐6 months), juvenile (6‐12 months), young adult (12‐24 months), mature adult (2‐6 years), senior (7‐11 years), and geriatric (>12 years).^17^ To achieve enough power in the statistical analysis, age subsequently was regrouped into dogs ≤2 years of age (puppies, juveniles, young adults) and dogs >2 years of age (mature adults, seniors, geriatrics).

Statistical analyses were performed using NCSS (NCSS 12 Statistical Software [2018], NCSS, LLC. Kaysville, Utah; ncss.com/software/ncss). Because of the low number of animals per group, we used the nonparametric Mann‐Whitney *U* test to test for differences in numerical variables (CSF peak velocity as well as numerical age and body weight). A Chi‐squared test was used to test for differences between 2 categorical variables, such as successful measurement (yes/no) and sex. In addition, associations between CSF peak flow velocity and numerical age and body weight were analyzed using Pearson correlation coefficients and linear regression models.

## 3 RESULTS

At the mesencephalic aqueduct, CSF peak flow velocity was determined successfully in 27 dogs (84.4%). At the FM, measurements were successful in 29 dogs (90.6%) within the ventral SAS and in 20 dogs (62.5%) within the dorsal SAS. Concerning the level of C2, 19 (59.4%) successful measurements within the ventral SAS and 20 (62.5%) within the dorsal SAS of C2 were achieved. In 12 dogs (37.5%), CSF flow quantification was successful at all 3 locations. Those dogs had a mean age of 3.28 years (median, 1.73 years; range, 0.66‐9.1 years), a mean body weight of 22.1 kg (median, 20.25 kg; range, 4‐51 kg), and included 10 (83.3%) females and 2 (16.7%) males. In 1 dog, a 6.3‐year‐old male Border Collie with a body weight of 23 kg, no CSF velocity measurements were achieved at any location. In 20 (62.5%) dogs, single CSF peak flow velocity measurements were unsuccessful. Dogs had a mean age of 4.21 years (median, 2.54 years; range, 0.35‐11.5 years), a mean body weight of 25.53 kg (median, 25.5 kg; range, 6.8‐53 kg), and included 6 (30%) females and 14 (70%) males. No evidence of difference in age (Mann‐Whitney *U* test; *P* = .61) and body weight (Mann‐Whitney *U* test; *P* = .28) between the dogs with individual nonsuccessful measurements and the dogs with fully successful measurements was found. However, dogs with fully successful CSF peak flow measurements contained a higher proportion of females (Chi‐squared test; *P* = .004).

The mean CSF peak flow velocities of the 3 locations are presented in Table 1. The lowest CSF peak flow velocities were recorded in the mesencephalic aqueduct and the highest in the dorsal SAS of C2. To avoid aliasing, PC‐MRI scans were repeated with a VENC setting up to 5 cm/s at the mesencephalic aqueduct and 7 cm/s at the FM and C2.

**TABLE 1:** Mean CSF peak flow velocity at the different locations in all dogs and grouped for body weight, age, and sex.

	Mesencephalic aqueduct	FM		C2	
		Ventral SAS	Dorsal SAS	Ventral SAS	Dorsal SAS
*All dogs*					
Mean CSF peak flow velocity ± SD (cm/s)	0.85 ± 0.48	2.05 ± 1.06	1.72 ± 0.77	2.37 ± 0.84	2.49 ± 1.13
n	27	29	20	19	20
*Dogs < 10 kg*					
Mean CSF peak flow velocity ± SD (cm/s)	0.64 ± 0.30	1.37 ± 0.36	0.92 ± 0.18	1.44 ± 0.39	1.34 ± 0.15
n	4	5	3	4	4
*Dogs between 10 and 20 kg*					
Mean CSF peak flow velocity ± SD (cm/s)	0.72 ± 0.33	2.07 ± 1.37	1.63 ± 0.81	2.47 ± 0.87	2.61 ± 1.46
n	7	8	6	5	6
*Dogs > 20 kg*					
Mean CSF peak flow velocity ± SD (cm/s)	0.96 ± 0.56	2.25 ± 1.0	1.98 ± 0.72	2.69 ± 0.72	2.88 ± 0.85
n	16	16	11	10	10
*Puppies (0–6 months)*					
Mean CSF peak flow velocity ± SD (cm/s)	1.1 ± 0.25	3.52 ± 2.50	2.13 ± 1.43	‐	5.32
n	2	2	2	0	1
*Juveniles (6–12 months)*					
Mean CSF peak flow velocity ± SD (cm/s)	0.72 ± 0.22	1.72 ± 0.15	1.23 ± 0.51	2.34 ± 0.82	1.95 ± 0.79
n	2	2	2	2	2
*Young adults (12–24 months)*					
Mean CSF peak flow velocity ± SD (cm/s)	0.81 ± 0.35	2.31 ± 1.11	1.91 ± 0.74	2.88 ± 0.92	2.72 ± 1.06
n	9	12	8	8	9
*Mature adults (2–6 years)*					
Mean CSF peak flow velocity ± SD (cm/s)	0.74 ± 0.25	1.52 ± 0.48	1.52 ± 0.25	1.94 ± 0.59	2.14 ± 0.77
n	7	7	4	7	6
*Seniors (7–11 years)*					
Mean CSF peak flow velocity ± SD (cm/s)	0.98 ± 0.83	1.76 ± 0.71	1.57 ± 1.10	1.84 ± 0.30	1.6 ± 0.27
n	7	6	4	2	2
*Female dogs*					
Mean CSF peak flow velocity ± SD (cm/s)	0.70 ± 0.22	1.62 ± 0.44	1.51 ± 0.66	2.22 ± 0.68	2.02 ± 0.57
n	15	15	12	12	12
*Male dogs*					
Mean CSF peak flow velocity ± SD (cm/s)	1.04 ± 0.65	2.50 ± 1.33	2.02 ± 0.86	2.63 ± 1.07	3.19 ± 1.41
n	12	14	8	7	8

Abbreviations: n, number of successful measurements; SD, standard deviation.

### 3.1 Influence of body weight on CSF peak flow velocity

**FIGURE 2:**
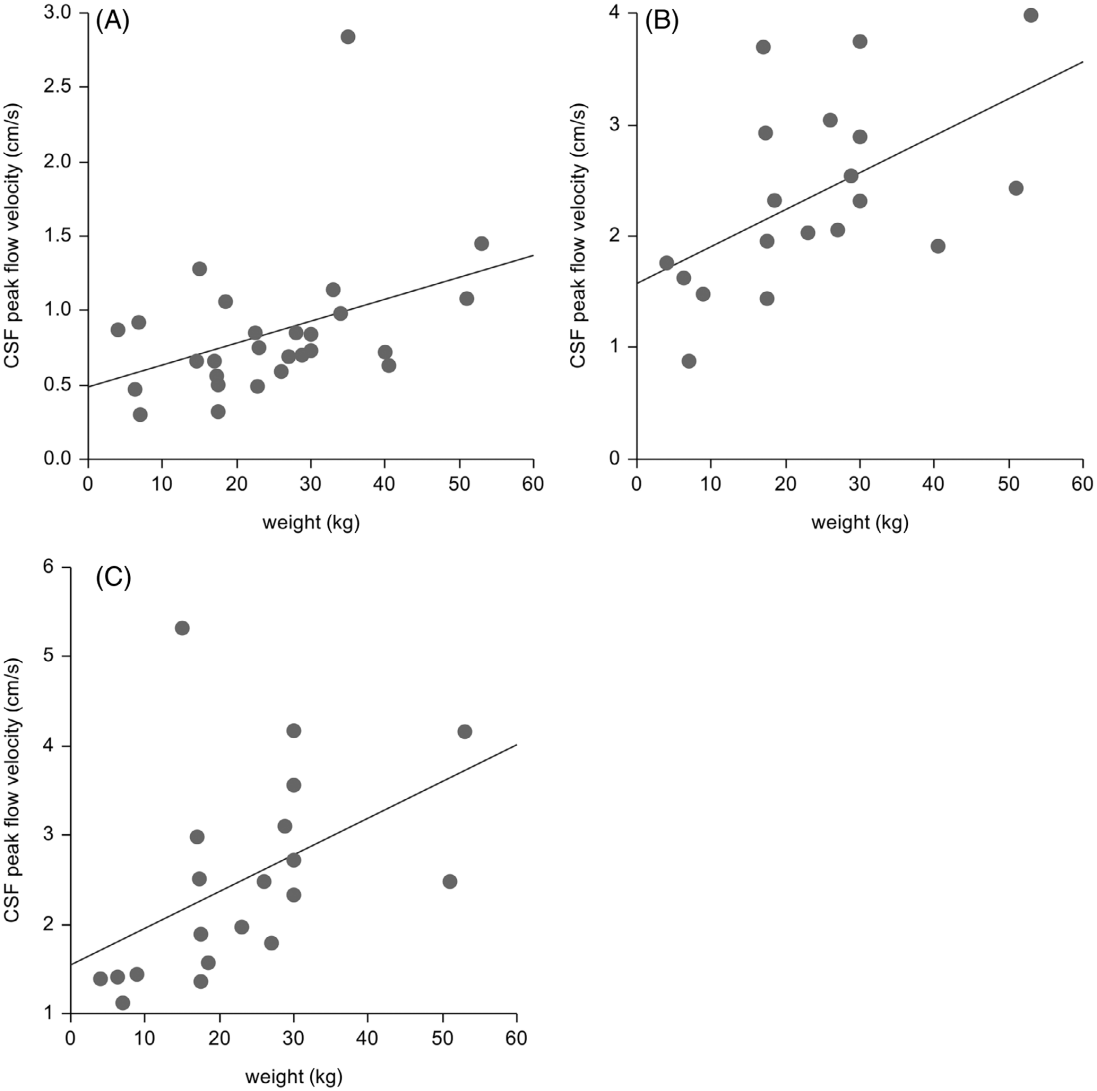
Scatter plots and linear regression models of (A) the mesencephalic aqueduct (AQ) (Estimated model: AQ velocity = 0.48 + 0.01 weight (kg); *P* = 0.04); (B) ventral subarachnoidal space (SAS) of the second vertebral body (C2) (Estimated model: ventral C2 velocity = 1.58 + 0.03 weight (kg); *P* = 0.02) and (C) dorsal SAS of C2 (Estimated model: dorsal C2 velocity = 1.54 + 0.04 weight (kg); *P* = 0.03).

**FIGURE 3:**
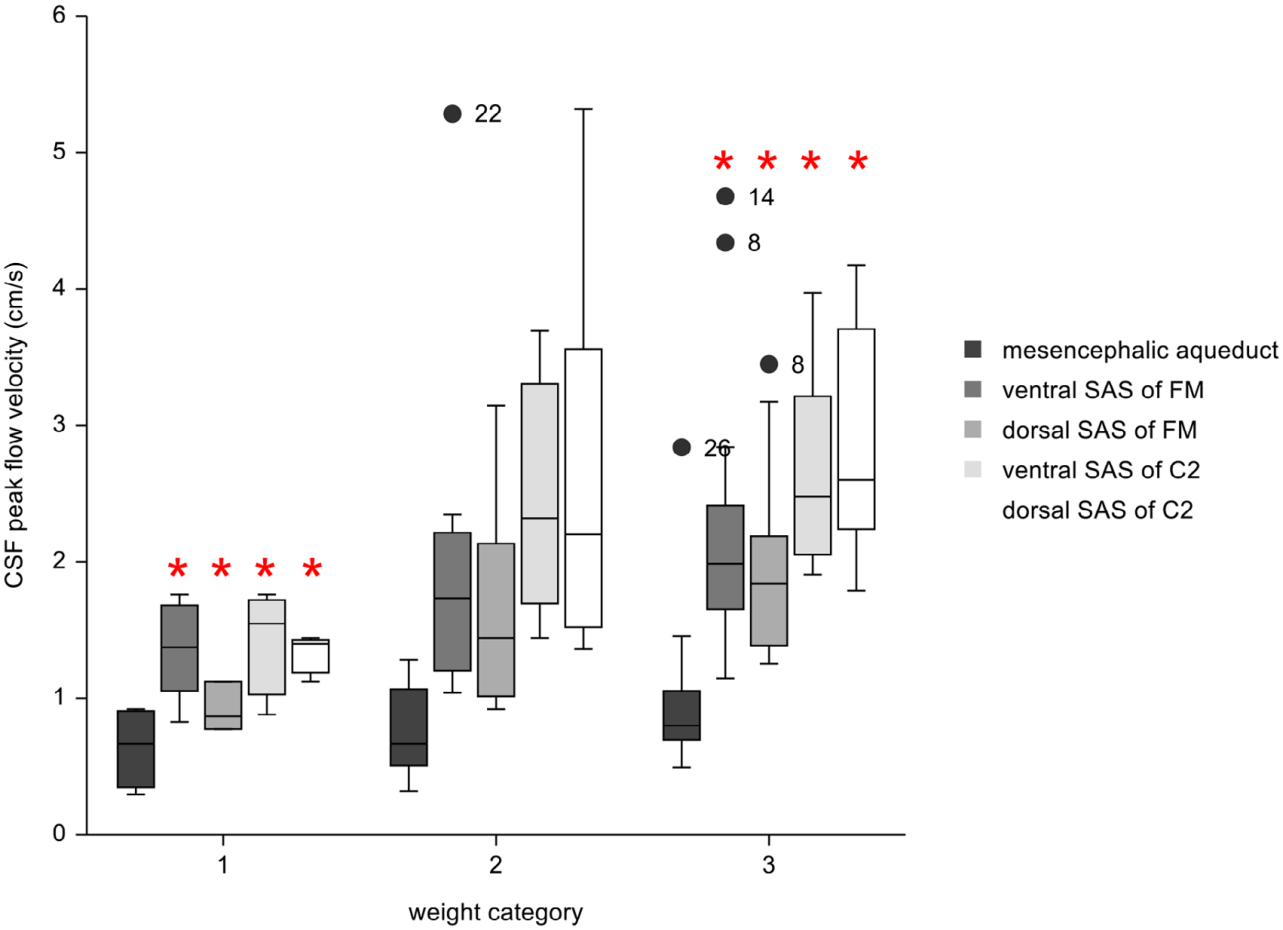
Box and Whiskers plots of CSF peak velocities at the different locations and according to different weight categories. Category 1: body weight <10 kg; category 2: body weight between 10 and 20 kg; category 3: body weight >20 kg. The length of the whiskers is 1.5 times the interquartile range. Observations larger than this distance are considered as outliers: Great Swiss Mountain Dog (dog number 8 and 14), American Bulldog (dog number 22), American Staffordshire Terrier (dog number 26). Within each of the body weight groups 1 and 3 the red stars indicate significant differences in CSF peak flow velocity between those locations.

Higher CSF peak flow velocity with higher body weight was found at the mesencephalic aqueduct (Pearson correlation = 0.39; *P* = .04) and the ventral SAS (Pearson correlation = 0.54; *P* = .02) and dorsal SAS (Pearson correlation = 0.48; *P* = .03) of C2 (Figure 2). Weak or no evidence of higher CSF peak flow velocity with higher body weight was found within the ventral SAS (Pearson correlation = 0.34; *P* = .07) and dorsal SAS (Pearson correlation = 0.34; *P* = .15) of the FM. Grouping of dogs according to body weight resulted in 5 dogs <10 kg, 8 dogs weighing between 10 and 20 kg, and 19 dogs weighing >20 kg. Dogs >20 kg of body weight had a significantly higher CSF peak flow velocity compared with dogs <10 kg of body weight at the ventral (Mann‐Whitney *U* test, *P* = .02) and dorsal (Mann‐Whitney *U* Test; *P* = .01) SAS of the FM, ventral SAS (Mann‐Whitney *U* test; *P* = .005) and dorsal SAS (Mann‐Whitney *U* test; *P* = .005) of C2 (Figure 3).

In our sample, dogs with body weight between 10 and 20 kg were significantly younger compared with dogs weighing >20 kg (Mann‐Whitney *U* test; *P* = .03). There was no evidence of a difference in age between dogs <10 and >20 kg body weight (Mann‐Whitney *U* test; *P* > .05). Dogs both <10 kg and between 10 and 20 kg of body weight were represented, with approximately 60% females and 40% males. Of the dogs >20 kg of body weight, 42% were females and 58% were males. No significant differences were found between the sexes in the different weight categories (Chi‐squared test, *P* = .14).

### 3.2 Influence of age on CSF peak flow velocity

No evidence of a significant correlation was found between numerical age and CSF peak flow velocity at any location. At the ventral (Pearson correlation = −0.40; *P* = .09) and dorsal (Pearson correlation = −0.39; *P* = .09) SAS of C2, weak evidence of lower CSF peak velocity with higher age was found.

**FIGURE 4:**
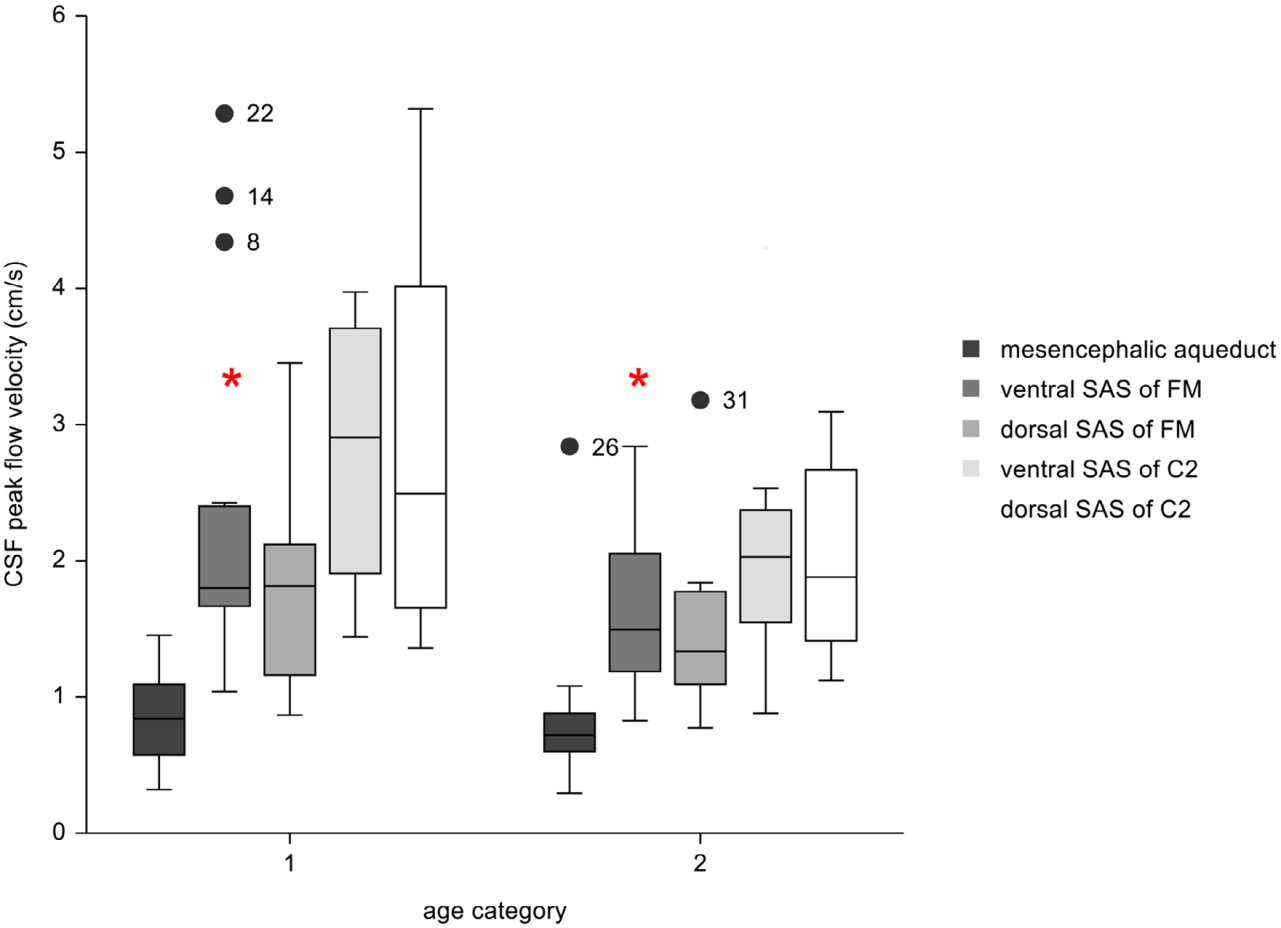
Box and Whiskers plots of CSF peak velocities at the different locations and according to different age categories. Category 1: dog ≤2 years; category 2: dog >2 years. The length of the whiskers is 1.5 times the interquartile range. Observations larger than this distance are considered as outliers: Great Swiss Mountain Dog (dog number 8 and 14), American Bulldog (dog number 22), American Staffordshire Terrier (dog number 26), Briard (dog number 31). Within each of the age groups the red stars indicate significant differences in CSF peak flow velocity.

Compared with dogs >2 years of age (mature adults, seniors, geriatrics), evidence of higher CSF peak flow velocity at the ventral SAS of the FM (Mann‐Whitney *U* test, *P* = .05) was found in dogs ≤2 years of age (puppies, juveniles, young adults; Figure 4). No evidence of difference was found between both groups with respect to body weight (Mann‐Whitney *U* test; *P* = .6) or sex because each group contained similar proportions of each sex.

### 3.3 Influence of sex on CSF peak flow velocity

**FIGURE 5:**
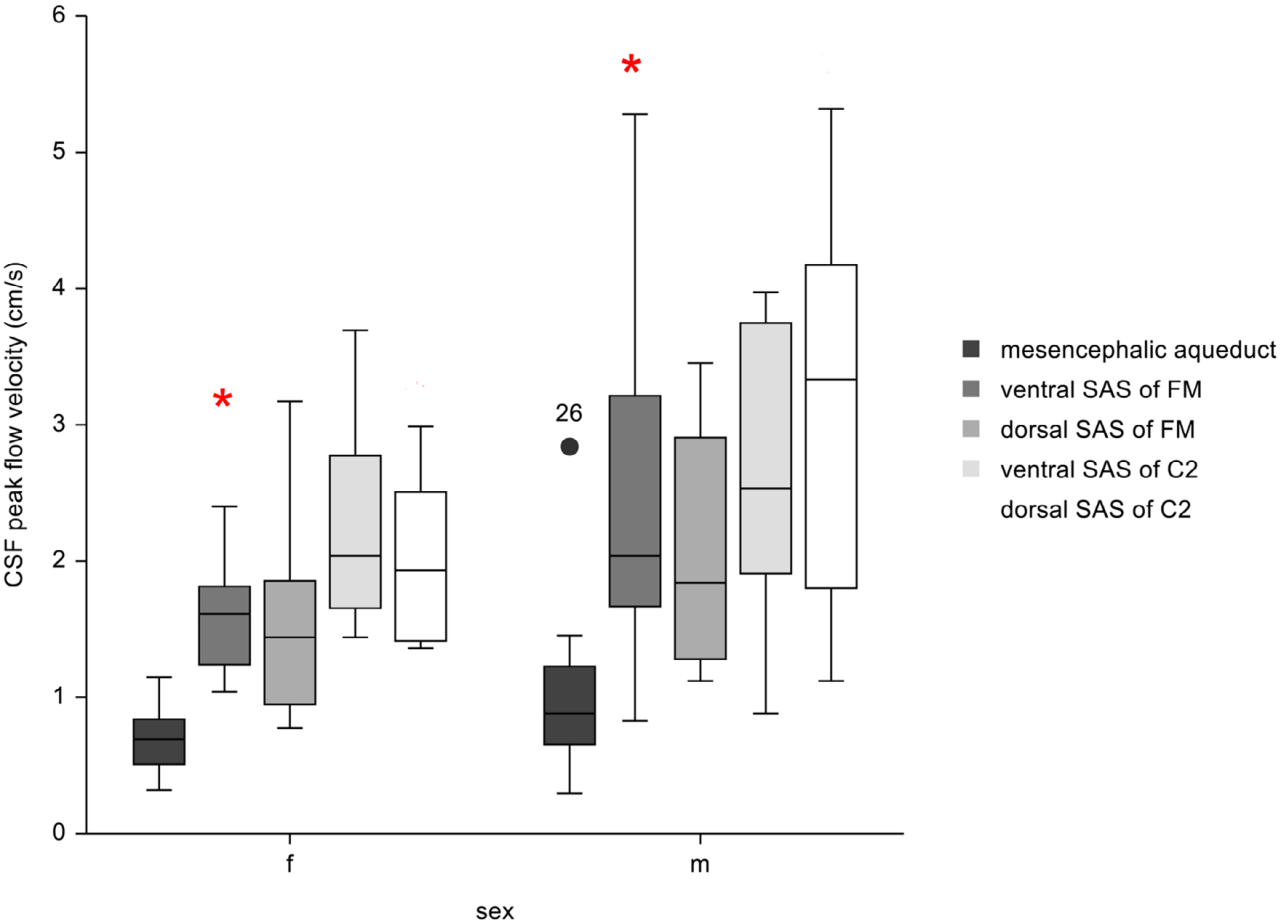
Box and Whiskers plots of CSF peak velocities at the different locations assigned to their sex. f, female dogs; m, male dogs. The length of the whiskers is 1.5 times the interquartile range. Observations larger than this distance are considered as outliers: American Staffordshire Terrier (dog number 26). Within each of the sex the red stars indicate significant differences in CSF peak flow velocity.

Significantly lower CSF peak flow velocity within the ventral SAS of the FM (Mann‐Whitney *U* test; *P* = .04) was found in females compared with males (Figure 5). Peak CSF flow velocities within the mesencephalic aqueduct (Mann‐Whitney *U* test; *P* = .08), the dorsal SAS of the FM (Mann‐Whitney *U* test; *P* = .16), and the ventral (Mann‐Whitney *U* test; *P* = .24) and dorsal (Mann‐Whitney *U* test, *P* = .06) SAS of C2 were not significantly different. No difference was found in body weight (Mann‐Whitney *U* test; *P* = .28) and age (Mann‐Whitney *U* test; *P* = 1.00) between females and males.

## 4 DISCUSSION

Different signalment characteristics in dogs influence CSF flow velocity. Higher CSF peak flow velocities were found in heavier dogs, in dogs ≤2 years of age, and in male dogs.

At all 3 anatomical locations, mean CSF peak flow velocity was the highest in dogs with body weight >20 kg. A correlation between CSF peak flow velocity and body weight in humans is not reported. But compared with fully grown dogs, where breed differences are associated with substantial size variability, the variation in body weight in adult humans is smaller. Possibly, this difference explains why, in dogs, an influence of body weight is seen. The dependency of CSF flow velocity and body weight also might explain why CSF peak flow velocities measured in dogs are in general much lower compared with humans.^18^, ^19^, ^20^, ^21^ Other reasons for the lower CSF peak velocities in dogs compared with humans also could be the difference between bipeds and quadrupeds or the difference in skull shape.

Peak CSF flow velocity of male dogs was higher at all 3 locations compared with female dogs, with significant differences within the ventral SAS of the FM. Because no significant difference was found in mean body weight and age between males and females, the sex of dogs affects CSF peak flow velocity. Such an influence of sex on CSF peak velocity within the cerebral aqueduct also is known in humans and could be explained by sex‐dependent neural and hormonal differences between females and males.^12^, ^13^

Higher CSF peak flow velocities at the ventral SAS of the FM were identified in dogs ≤2 years of age. Age‐related differences in CSF peak velocity are reported in healthy humans. Higher CSF peak velocities at the FM were observed in pediatric patients between 3 and 16 years compared with adults between 21 and 61 years of age.^11^ This age‐dependent decrease of CSF peak velocity at the FM is observed mainly in the first 2 decades of life, with little changes thereafter.^11^ At the cerebral aqueduct, CSF peak velocity is higher in children (1‐10 years) compared with adolescents (10‐17 years), with no difference in infants (1‐12 months).^13^ In contrast, between 17 and 88 years of age, CSF peak velocity within the cerebral aqueduct increases with every year by 0.031 cm/s.^12^ Such a pattern was not observed in our study. The cause for age‐related changes in CSF flow velocity is unknown. Some hypothetical explanations are larger displacement of CSF by the arterial pressure wave in children,^11^ or differences in parenchymal compliance, blood volume, CSF volume, and the cardiac cycle between children and adults.^19^, ^22^

We did not find evidence of a significant correlation between CSF peak velocity and age in dogs. However, our study group only included 4 dogs <1 year of age that might correspond to pediatric human patients. One of them, a 5‐month‐old American Bulldog (dog number 22), showed much higher CSF peak flow velocities. Because the CSF peak flow velocities of the other 3 pediatric dogs were not that high, pediatric age is unlikely the cause of the high CSF peak flow velocities. It seems more likely that other factors not investigated were responsible for the high CSF velocities in this dog.

The statistical outliers indicate additional influences on CSF flow velocity. The number of brachycephalic dogs in our study group was low (n = 4). Therefore, a comparison of CSF peak flow velocity among brachycephalic, mesocephalic, and dolichocephalic dogs was not done. An 8‐year‐old male American Staffordshire Terrier (dog number 26) also showed high CSF peak velocity at the mesencephalic aqueduct and ventral SAS of the FM. In 2 other brachycephalic dogs, a 6‐year‐old male American Staffordshire Terrier and a 5‐year‐old female Yorkshire Terrier, the CSF peak flow velocities were within the range of the individual weight, age, and sex classification. To identify a possible influence of skull conformation, CSF flow measurement in a higher number of dogs with different cephalic indexes is needed.

The number of successful CSF peak flow velocity measurements was lower compared with existing data in dogs.^5^, ^9^, ^10^ A lack of phase contrast signal occurred especially within the dorsal SAS at the FM and within the ventral and dorsal SAS at C2. Possible causes are the difficulty in measuring CSF flow in small CSF spaces and the lower CSF peak flow velocities in dogs compared with humans.^11^, ^16^, ^23^ Both result in a low signal‐to‐noise ratio, which is more severe using a weaker magnet. The significantly higher proportion of females in dogs with individual unsuccessful CSF peak velocity measurements indicates that low CSF flow velocities are a possible reason. However, mean CSF peak flow velocities in our study were slightly higher compared with those of previous studies done using different magnetic field strengths, anesthesia, and examination protocols (Table 2).^5^, ^9^, ^10^

**TABLE 2:** Listing of the mean CSF peak flow velocity ± SD at the different locations in all studies where CSF flow was measured by PC‐MRI in healthy dogs.

			Cerda‐Gonzalez et al.^5^	Christen et al.^9^	Cho et al.^10^	Current study
Magnetic field strength (T)			1.5	3	1.5	1
VENC setting (cm/s)			2–4	10	8	3–5
Mean CSF peak flow velocity ± SD (cm/s)	Aqueduct			0.92 ± 0.5	0.76 ± 0.43	0.85 ± 0.48
	FM	Ventral SAS	0.75 ± 0.24	Left: 1.9 ± 0.8 Right: 1.78 ± 0.7	1.39 ± 0.13	2.05 ± 1.06
		Dorsal SAS	0.59 ± 0.13	1.17 ± 0.4	0.32 ± 0.12	1.72 ± 0.77
	Cervical spine	Ventral SAS	0.29	Left. 1.91 ± 0.5 Right: 2.15 ± 0.8		2.37 ± 0.84
		Dorsal SAS	0.66	1.27 ± 0.4		2.49 ± 1.13

Another reason for the low number of successful measurements could be the positioning of the dogs. In our study, the dogs were positioned with an extended head‐neck position as was done previously.^9^, ^10^ The size of the CSF space of the SAS depends on the head‐neck position during the MRI examination.^5^ A mildly flexed head position resulted in a higher number of successful measurements in the dorsal SAS of the FM and is therefore important for optimal use in dogs.^5^

Other possible reasons for nonsuccessful CSF flow measurements are inaccurate placement of the measurement plane or an inadequate VENC setting.^4^ The VENC is a crucial parameter of the PC‐MRI examination. Aliasing artifacts are caused by underestimating the VENC value, whereas overestimation of the VENC leads to low signal.^3^ The advice is to select a VENC that is slightly above the flow velocity of the fluid.^3^, ^24^ The VENC settings were therefore consistently controlled for the patients and adapted, if necessary. The VENC settings of our study usually were lower compared with previous studies (Table 2).^5^, ^9^, ^10^

Our sample recruited client‐owned dogs, presented to the clinic for low‐risk diagnostic procedures such as radiography for hip and elbow dysplasia. This majority of young adult, large breed dogs with medium body weight may not represent the larger population of dogs in Switzerland. Future studies should aim to recruit a higher number of small‐breed dogs with different skull conformations. Furthermore, brachycephalic, small‐breed dogs belong to the population with the highest risk for diseases with CSF flow alterations, such as Chiari‐like malformations and hydrocephalus.^25^

## 5 LIMITATIONS AND STRENGTHS

One limitation of our study is that CSF peak flow velocity was compared with body weight as a marker of dog size. However, body weight varies with nutritional status and body conformation. Therefore, it does not reliably reflect the size of the dogs. Possibly, the length of the spine or height of the dog might be more reliable and should be used in future studies.

General anesthesia influences CSF peak flow velocity. It is known that in humans under general anesthesia hypercapnia (end‐tidal CO_2_ = 60 mm Hg) increases systolic CSF peak velocity in the aqueduct of Sylvius compared with normocapneic humans (end‐tidal CO_2_ = 40 mm Hg).^26^ To minimize this influence, dogs in our study were mechanically ventilated to achieve an end‐tidal CO_2_ between 35 and 40 mm Hg. The use of dexmedetomidine and anesthesia has the potential to influence hemodynamic and respiratory variables,^27^ as well as the measurements of our study. However, anesthesia is necessary to perform MRI examination in dogs. The protocol was chosen because it is believed to be clinically relevant.

A further limitation of our study is the exclusion of measurements because of a lack of bright‐to‐dark shifts in the CSF space. As a result, potentially excessively low CSF peak flow velocities were excluded. This missing data might have influenced the CSF peak velocities toward false increases in mean CSF peak flow velocity.

## 6 CONCLUSION

We showed that quantification of CSF flow varies with sex, body weight, and age when determined by PC‐MRI in dogs without neurological disorders. Peak CSF flow velocity is higher in males compared with female dogs. Furthermore, CSF peak flow is higher in dogs with higher body weight, suggesting that large breed dogs have higher CSF peak flow velocity compared with small breed dogs. Finally, dogs ≤2 years of age have higher CSF peak flow velocity. Sex, body weight, and age must be considered in dogs, when CSF flow is quantitatively assessed.

## CONFLICT OF INTEREST DECLARATION

Authors declare no conflict of interest.

## OFF‐LABEL ANTIMICROBIAL DECLARATION

Authors declare no off‐label use of antimicrobials.

## INSTITUTIONAL ANIMAL CARE AND USE COMMITTEE (IACUC) OR OTHER APPROVAL DECLARATION

Approved by the Cantonal Veterinary Office of Bern TVB Nr.: BE 4/18 according to the Swiss national animal protection law.

## HUMAN ETHICS APPROVAL DECLARATION

Authors declare human ethics approval was not needed for this study.
